# Larvicidal and Immunomodulatory Effects of Conidia and Blastospores of *Beauveria bassiana* and *Beauveria brongniartii* in *Aedes aegypti*

**DOI:** 10.3390/jof11080608

**Published:** 2025-08-21

**Authors:** José L. Ramirez, Haley M. Gore, Angela Payne, Salorrane Miranda Nascimento Pinto, Lina B. Flor-Weiler, Everton K. K. Fernandes, Ephantus J. Muturi

**Affiliations:** 1 Crop BioProtection Research Unit, USDA-ARS, National Center for Agricultural Utilization Research, 1815 N. University St., Peoria, IL 61604, USA; 2 Instituto de Patologia Tropical e Saúde Pública, Universidade Federal de Goiás, Goiânia 74690-900, GO, Brazil

**Keywords:** microbial biopesticide, mycopesticide, fungal propagule, fungal biopesticides, microbial control

## Abstract

The increasing global burden of mosquito-borne diseases and the widespread development of insecticide resistance in mosquitoes have fueled renewed interest in entomopathogenic fungi as effective tools that are compatible with existing mosquito control strategies. These fungi produce different types of infective propagules, including hydrophobic conidia and yeast-like blastospores, which differ in structure, mode of infection, and virulence. In this study, we evaluated the larvicidal activity of conidial and blastospore propagules from *Beauveria bassiana* MBC076 and *Beauveria brongniartii* MBC397 against *Aedes aegypti*. Conidia exhibited more rapid and more potent larvicidal effects compared to blastospores, but the overall survival at seven days post-infection was similar between the two types of propagules. Interestingly, *B. brongniartii* blastospore infections resulted in a significantly higher proportion of pupal mortality, suggesting a delayed mode of action. Immune profiling of infected larvae indicated significant induction of antimicrobial effectors such as cecropin, defensin, and attacin, primarily in response to conidial infection. In contrast, blastospore infections were associated with reduced expression of several prophenoloxidase genes, particularly during infection with *B. brongniartii* blastospores. These findings indicate that different fungal species and their propagule types exert varying levels of virulence and immune modulation in mosquito larvae. This study provides insights into the infection dynamics of fungal propagules and identifies immune markers that can be leveraged to enhance the efficacy of fungal-based larvicides.

## 1. Introduction

The growing burden of mosquito-borne diseases, coupled with the widespread emergence of insecticide resistance, has intensified the search for integrated compatible alternatives that can complement existing chemical control strategies. Fungal entomopathogens are increasingly recognized as valuable tools within integrated pest management tools because they are ecofriendly, highly effective at controlling insects, and can often circumvent insecticide resistance mechanisms [1,2,3,4,5,6]. To initiate a fungal infection in an insect, fungal conidia attach to the insect cuticle, germinate, and penetrate the insect’s body cavity (hemocoel), often forming hyphal bodies or blastospores which can lead to insect death [6,7].

Fungal conidia and blastospores are distinct fungal propagules that substantially differ in structure and virulence. Conidia are hydrophobic and environmentally resilient spores, while blastospores are yeast-like cells produced inside the insect hemocoel and are better adapted for rapid proliferation and immune evasion [8,9,10,11]. For application purposes, conidia are usually grown in solid media while the thin-walled blastospores are produced during a short fermentation process [6,8,12]. These two propagule types can differ in virulence depending on the fungal strain, isolate, dose, and insect host [2,9,13,14]. Although, fungal blastospores tend to be more virulent than conidia [8,9], some strains of fungal conidia can be more virulent than their blastospore counterpart in the aquatic environment [2,15,16,17].

Mosquito susceptibility to fungal infection is strongly influenced by local and systemic immune responses [18,19,20,21,22]. In adult mosquitoes, antifungal immunity involves canonical innate signaling cascades such as the Toll and Immune Deficiency (IMD) pathways [7,23,24], which activate the transcription of effector molecules including antimicrobial peptides (AMPs) and lysozymes [22,25]. These peptides, acting synergistically, disrupt fungal cell membranes and interfere with fungal physiology, a step critical for controlling disseminated fungal infections [20,25,26].

Another essential component of the mosquito antifungal arsenal is the melanization response, a humoral defense mediated by the enzymatic activation of prophenoloxidase (PPO) into phenoloxidase (PO) [27,28]. The melanization cascade encapsulates and neutralizes invading pathogens and has been shown to limit fungal proliferation and virulence [27,29]. However, some entomopathogenic fungal species appear capable of evading or suppressing this response [7,26,29]. Adding to this, the two fungal propagule forms are not only morphologically distinct but may also elicit differential host immune responses, a critical component of microbial biopesticide efficiency.

While less is known about mosquito larval responses to fungal entomopathogens, studies have shown a pattern similar to the adult stage. For instance, infections with *Metarhizium brunneum* blastospores lead to increases in AMP expression in *Aedes aegypti* [8], while exposure to conidia or blastospores from the same fungus increased phenoloxidase activity in *Culex* larvae [16]. In addition, a study by Bitencourt et al. [9] described an increase in PO activity and suppression of AMPs in *Ae. aegypti* infections with *Beauveria bassiana*. Thus, complex interactions seem to govern mosquito antifungal responses that appear to be dependent on the type of infecting propagule.

In this study, we evaluated the virulence of two fungal entomopathogens, *B. bassiana* and *B. brongniartii*, applied as either conidia or blastospores against *Ae. aegypti* larvae. We quantified larval survival and characterized larval immunity by measuring the expression of antimicrobial peptides and PPO isoforms, key components of the antifungal response. This study advances the field of microbial mosquito control by characterizing fungal propagules with higher virulence and by identifying immune biomarkers that can be utilized to optimize fungal biopesticides for vector control.

## 2. Materials and Methods

### 2.1. Mosquito Rearing

*Aedes aegypti* (Rockefeller strain, obtained from BEI Resources) mosquitoes were reared under standard rearing conditions at 28 °C, 12 h light/dark cycle, and 60–70% humidity. The larvae were provided a 2:1:1 rabbit food–liver powder–fish flakes diet. For egg production, mated adult females were offered a citrated bovine blood meal (Hemostat Laboratories, Inc., Dixon, CA, USA) via an artificial membrane feeding system (Chem Glass, Vineland, NJ, USA).

### 2.2. Fungal Cultures and Fungal Species

Two entomopathogenic fungal strains, *Beauveria bassiana* (MBC 076) and *Beauveria brongniartii* (MBC 397), were evaluated for their larvicidal activity against *Ae. aegypti*. Initial cultures were revived from glycerol stocks stored at –80 °C. To ensure optimal mycelial growth and conidia production, *B. bassiana* MBC 076 was cultured on ¼ strength Sabouraud dextrose agar supplemented with yeast extract (¼ SDAY), while *B. brongniartii* MBC 397 was cultured on Potato Dextrose Agar (PDA), as each species demonstrated optimal growth on these respective media during preliminary trials. Both fungi were incubated for a minimum of two weeks under a 12 h light/dark cycle at ambient temperature in a lightbox to promote sporulation.

Blastospores were produced by first suspending conidia to a concentration of 1 × 10^7^ conidia/mL in 10 mL of sterile water and subsequently inoculating this suspension into basal liquid medium supplemented with trace metals to a volume of 100 mL in baffled 250 mL flasks. Cultures were incubated at 28 °C, shaking at 350 rpm for three days. After incubation, cultures were strained through a No. 100 sieve (150 µm openings) to separate blastospores from hyphal fragments, and blastospores were collected by centrifugation at 1000× *g* for 10 min. The resulting pellet was resuspended in 5 mL of 0.2% Tween 20 (Sigma-Aldrich, St. Louis, MO, USA), and blastospore concentrations were determined using a Bright-line hemocytometer. Five serial 1:5 dilutions were prepared from an initial concentration of 1 × 10^9^ propagules/mL to a final concentration of 1.6 × 10^6^ propagules/mL for larval treatments.

Conidia inocula were prepared by scraping spores from agar plates with a sterile inoculation loop and suspending them in 1 mL of 0.2% Tween 20. Suspensions were homogenized by vortexing with a Disruptor Genie (Scientific Industries, New York, NY, USA) and filtered through two layers of sterile cheesecloth to remove mycelial debris. Conidial concentrations were quantified using a hemocytometer, and five serial 1:5 dilutions were prepared starting from 5 × 10^9^ propagules/mL down to 8 × 10^6^ propagules/mL for treatments.

### 2.3. Larvicidal Infection Bioassays

To evaluate the efficacy of the blastospore and conidia treatments, third-instar mosquito larvae (three days post-hatching) were counted into 6-well plates at a density of 10 larvae per well. Three replicate wells were prepared for each treatment. Each well contained 4 mL of deionized water, to which 0.5 mL of the appropriate blastospore or conidia suspension was added. Control groups consisted of three replicate wells receiving 0.5 mL of 0.2% Tween 20 solution instead of fungal inoculum. Each day larvae were fed a small amount of food and any dead larvae or dead pupae, if present, were removed.

Plates were maintained in an incubator at 26 °C with a 12 h light/dark cycle. Mortality was recorded daily, noting the developmental stage at the time of death (larval or pupal). At 7 days post-infection (dpi), the number of adults successfully emerging from their pupal exuviae (“survivors”) was recorded, along with the number of larvae and pupae that remained alive.

### 2.4. RNA Extraction and Gene Expression Analysis

For gene expression analysis, a separate cohort of larvae was infected with either conidia or blastospores at a concentration of 2 × 10^8^ spores/mL. Larvae were treated identically to those in the survival experiment. At 3 dpi, larvae were collected in pools of five individuals per treatment group. Larvae were homogenized in RLT buffer (RNeasy Mini Kit, Qiagen, Hilden, Germany) using 3.2 mm macerating beads and a TissueLyser II instrument (Qiagen, Germany), and RNA was extracted following the manufacturer’s protocol.

RNA concentrations were measured using a NanoDrop spectrophotometer (ThermoFisher Scientific, Walthman, MA, USA), and samples were normalized to 1 µg of total RNA for cDNA synthesis using the QuantiTect Reverse Transcription Kit (Qiagen, Germany). Quantitative PCR (qPCR) reactions (10 µL volume) were set up in duplicate, combining the synthesized cDNA with PowerUp SYBR Green Master Mix (Life Technologies, San Francisco, CA, USA) and gene-specific primers at a final concentration of 300 nM. Our qPCR expression assays examined the expression of Rel1, Rel2, Tep22, cecropin A, cecropin D, lysozyme C, C11, C10, B, attacin, gambicin, defensin A, C, holotricin, and prophenoloxidase genes PPO1 through PP10. Fungal load was measured by quantifying the expression of the 18S rRNA fungal gene. To assess fungal recognition by the mosquito immune system, a proxy for successful fungal infection, we evaluated the expression of TEP22, a mosquito antifungal effector found to be significantly expressed in response to entomopathogenic fungal infections [30]. qPCR assays were conducted on a QuantStudio 6 Flex Real-Time PCR System (Applied Biosystems, Foster City, CA, USA) using the manufacturer’s recommended cycling conditions: an initial holding stage at 95 °C for 20 s, followed by 40 cycles of 95 °C for 1 s and 60 °C for 20 s. A melt curve analysis was included at the end of the run. The Ribosomal protein gene Rps17 (AAEL004175) was used as the reference [31,32]), and the ΔΔCt method was used to analyze and compare gene expression levels [33] (Primer sequences used in this study are presented in Table S1.

### 2.5. Statistical Analysis

Survival data and gene expression results following fungal infections were analyzed using Prism 10 software (GraphPad, San Diego, CA, USA). Gene expression was evaluated via the Kruskal–Wallis test with Dunn’s multiple comparison post-test. Survival curves following infections were generated from five serial 1:5 dilutions from 1 × 10^9^ to 1.6 × 10^6^ propagules/mL for blastospore treatments and from five serial 1:5 dilutions ranging from 5 × 10^9^ to 8 × 10^6^ propagules/mL for treatments with conidia. Survival curves were analyzed using the Kaplan–Meier method, and differences between treatment groups were assessed with the Log-rank (Mantel–Cox) test. To compare pupal and larval mortality proportions within each treatment, we first employed the Shapiro–Wilk test to assess normality. The Wilcoxon test was applied within each treatment group if it was determined that the data were not normal; otherwise, a paired *t*-test was used to evaluate statistical significance between pupae and larvae mortality proportions. The LC_50_ values were estimated using the GLM-Probit model in R (version R 3.6.0). Mortality data were adjusted with Abbott’s correction, and concentrations were log-transformed (base 10). A Probit regression was fitted, with adjusted mortality as the response and log-transformed concentration as the predictor. LC_50_ was calculated as LC_50_ = 10^−β^_0/_^β^_1_, with 95% confidence intervals (CIs) derived using the delta method. Significance was assessed at *p* ≤ 0.05.

## 3. Results

### 3.1. Conidial Propagules from B. bassiana MBC076 and B. brongniartii MBC397 Are More Effective than Blastospores at Reducing Larval Survival of Ae. aegypti Mosquitoes

**Figure 1:**
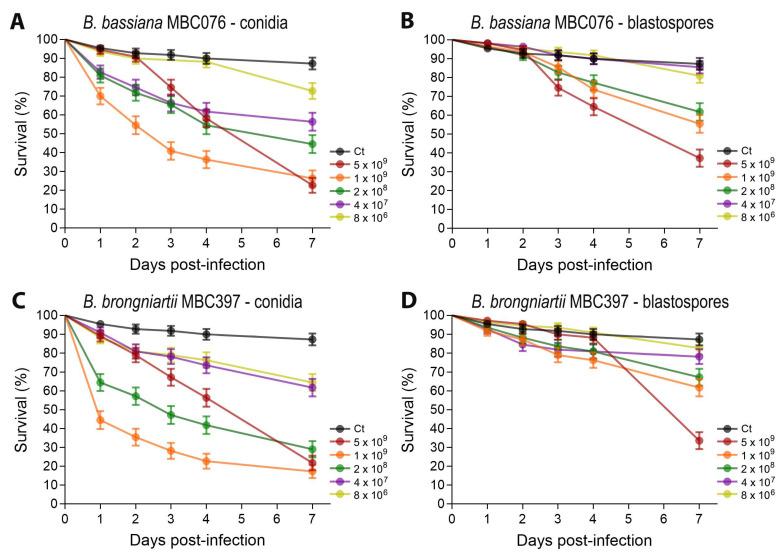
Survival curves following infection with either conidia (**A**,**C**) or blastospores (**B**,**D**) from *Beauveria bassiana* MBC076 and *Beauveria brongniartii* MBC397. Survival graphs represent 4 independent experiments, and data were analyzed via the log-rank Mantel–Cox test. Error bars indicate the SEM (GraphPad Prism 10).

To compare the virulence between conidial and blastospore propagules of *B. bassiana* MBC076 and *B. brongniartii* MBC397, we conducted dose–response larvicidal assays using five different doses. Mosquito survival varied significantly both between fungal species and between propagules of the same fungal species. For both fungal species, treatments with conidia generated the greatest impact on larval survival (*B. bassiana* MBC076: log-rank Mantel–Cox test: X^2^: 139.0, *p* < 0.0001, and *B. brongniartii* MBC397: log-rank Mantel–Cox test: X^2^: 200.5, *p* < 0.0001) compared to blastospores (*B. bassiana* MBC076: log-rank Mantel–Cox test: X^2^: 97.46, *p* < 0.0001, and *B. brongniartii* MBC397: log-rank Mantel–Cox test: X^2^: 74.71, *p* < 0.0001) (Figure 1).

Probit analysis revealed that conidia had significantly lower LC50 values compared to blastospores (Table 1). The median survival time (S50) upon exposure to 1 × 10^9^ conidia/mL for infections with *B. bassiana* MBC076 was higher (S50 = 3 days) than that observed for *B. brongniartii* MBC397 conidial infections (S50 = 1 day). Treatment with blastospores from either entomopathogenic fungi at the same propagule concentration generated similar median survival times (*B. bassiana* MBC076 S50 = 7 days; *B. brongniartii* MBC397 S50 = 7 days).

**Figure 2:**
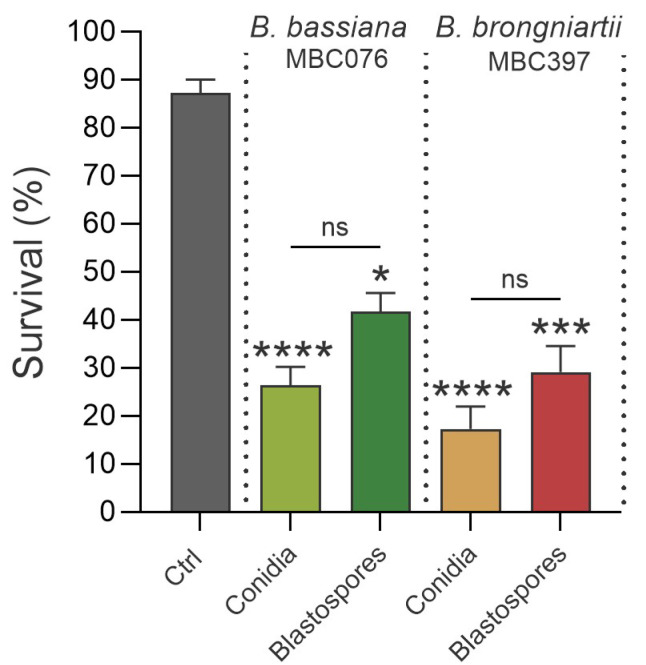
End-point mortality at 7 days post-infection following infection with either conidia or blastospores from *Beauveria bassiana* MBC076 and *Beauveria brongniartii* MBC397. Ctrl = Control group. Kruskal–Wallis test with Dunn’s multiple comparison post-test; * *p* < 0.05, *** *p* < 0.001, **** *p* < 0.0001, ns = not significant.

Conidial treatments reached median lethal times (S50) earlier than their corresponding blastospore treatments (Table 1), indicating a faster onset of mortality for conidial treatments despite some overlap in survival curves (Figure 2). Here, at 7 d post-exposure, while survival was lower in treatments with conidia, it was not significantly different from survival in larvae exposed to blastospores of the same fungi (Kruskal–Wallis test; *B. bassiana* MBC076: control vs. conidia *p* < 0.0001, control vs. blastospores *p* < 0.0351, conidia vs. blastospores *p* > 0.05; *B. brongniartii* MBC397: control vs. conidia *p* < 0.0001, control vs. blastospores *p* < 0.0003, conidia vs. blastospores *p* > 0.05) (Figure 2).

### 3.2. Conidial Propagules Primarily Kill at the Larval Stage, but Infection with B. brongniartii MBC397 Blastospores Exacts Significantly Greater Mortality at the Pupal Stage

**Figure 3:**
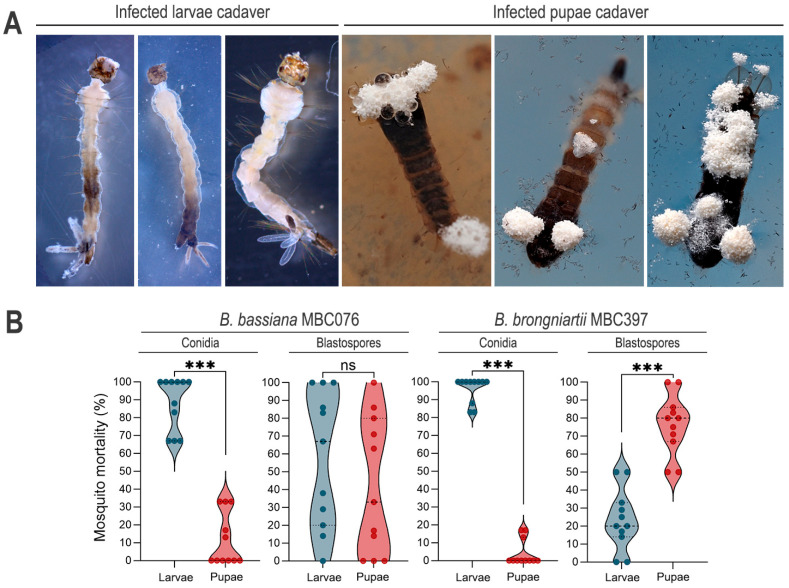
Stage-specific mortality following infection with either conidia or blastospores from *Beauveria bassiana* MBC076 and *Beauveria brongniartii* MBC397. (**A**) Larvae or pupae cadavers following entomopathogenic fungal infection. (**B**) Proportion of death by developmental stage (larvae vs. pupae). Control groups only presented a few deaths at the larval stage (not presented). Infections with blastospores were compared via paired *t*-test, while infections with conidia did not pass the normality test and were compared via Wilcoxon matched-pairs signed rank test; *** *p* < 0.001, ns = not significant.

Larval death presented a range of phenotypic characteristics, with some larvae presenting patches of melanotic pigmentation, primarily on those dying 24 h post exposure, while larvae with deaths occurring days after infection were white in appearance (Figure 3A). Dead pupae that floated on the water produced mycelia that emerged primarily from the respiratory trumpets and paddle vein that contacted the water surface. These mycelia sporulated readily, confirming successful infection of the mosquito larvae that carried into the pupal stage (Figure 3A). For both fungal species, mortality predominantly occurred during the larval stage in treatments with conidial propagules (*B. bassiana* MBC076 conidia, larvae vs. pupae: Wilcoxon matched-pairs test *p* < 0.001; *B. brongniartii* MBC397 conidia, larvae vs. pupae: Wilcoxon matched-pairs test *p* < 0.001) (Figure 3). However, while *B. bassiana* blastospores caused similar proportions of dead larvae and dead pupae (paired *t*-test, *p* = 0.513), treatment with *B. brongniartii* blastospores resulted in significantly higher mortality at the pupal stage than at the larval stage (Paired *t*-test, *p* = 0.0004) (Figure 3B).

### 3.3. Increased Fungal Loads in Larvae Exposed to Fungal Propagules and Induction of Fungal Infection Markers

**Figure 4:**
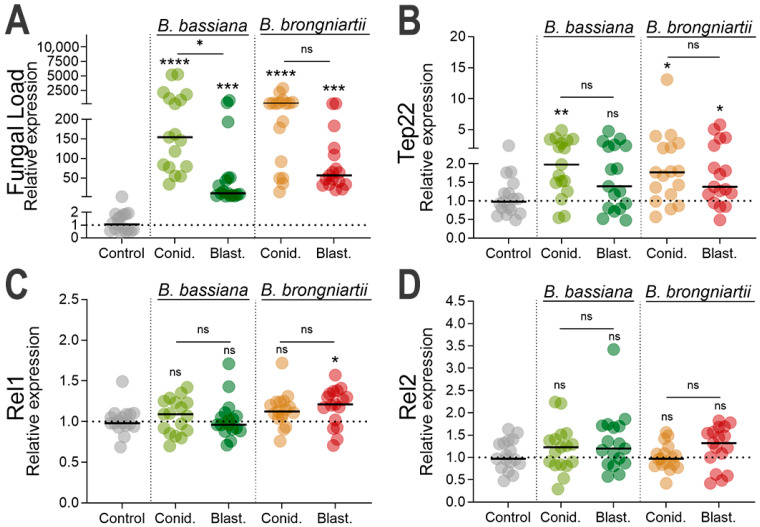
Fungal load and immune gene expression in mosquito larvae 3 days post-infection with *B. bassiana* or *B. brongniartii*. (**A**) Fungal loads determined by relative quantification of fungal 18S rRNA in whole-body larvae. Each dot represents the fungal load value from a pool of 5 mosquitoes, and the horizontal bar indicates the median fungal load. (**B**) Relative expression of the fungal recognition and fungal effector gene TEP22, (**C**) Relative expression of the transcription factor REL1 and (**D**) Relative expression of the transcription factor REL2. For all panels, larvae were exposed to conidia (Conid.) or blastospores (Blast.) of *B. bassiana* or *B. brongniartii*. Expression values are shown as fold change relative to control larvae. Data represent three independent experiments. Statistical significance was determined by Kruskal–Wallis test with Dunn’s post-test; * *p* < 0.05, ** *p* < 0.01, *** *p* < 0.001, **** *p* < 0.0001, ns = not significant.

To confirm successful larval infection by the two fungal entomopathogens, we evaluated fungal loads in whole mosquito bodies at three days post infection by analyzing the transcript abundance of the fungal 18S rRNA gene using fungi-specific primers [34] Our results indicated a significantly greater number of fungal genomes in larvae exposed to either *B. bassiana* MBC076 (conidia: *p* < 0.0001, blastospores: *p* = 0.0006) or *B. brongniartii* MBC397 (conidia: *p* < 0.0001, blastospores: *p* = 0.0002) in comparison to uninfected controls (Figure 4A). A significantly greater number of conidia fungal transcripts were observed in larvae infected with *B. bassiana* MBC076 conidia compared to larvae infected with blastospores (Kruskal–Wallis test, *p* = 0.0346). While similar trends were observed in larvae infected with *B. brongniartii* MBC397, it was not statistically significant (Kruskal–Wallis test, *p* = 0.1456) (Figure 4A).

TEP22 was significantly overexpressed in mosquito larvae infected with conidia of each of the two fungal species (*B. bassiana* MBC076: Kruskal–Wallis test, *p* = 0.0098 or *B. brongniartii* MBC397: Kruskal–Wallis test, *p* = 0.0227) (Figure 4B). Similarly, TEP22 was significantly overexpressed in mosquito larvae infected with *B. brongniartii* MBC397 blastospores (Kruskal–Wallis test, *p* = 0.0448) relative to the controls, but this effect was not found in mosquito larvae infected with *B. bassiana* MBC076 blastospores (Kruskal–Wallis test, *p* = 0.2506) (Figure 4B). We also evaluated the expression of REL1 and REL2, transcription factors of the Toll and IMD pathways, respectively, with assays indicating a slight but significant expression of REL1 in larvae infected with *B. brongniartii* MBC397 blastospores (Kruskal–Wallis test, *p* = 0.0223) relative to the controls. No other group showed a statistically significant increase relative to uninfected controls (Figure 4C,D).

### 3.4. Cecropin, Defensin, and Attacin Were the Most Significantly Induced Antimicrobial Effectors as Part of the Larval Resistance to Entomopathogenic Fungi

**Figure 5:**
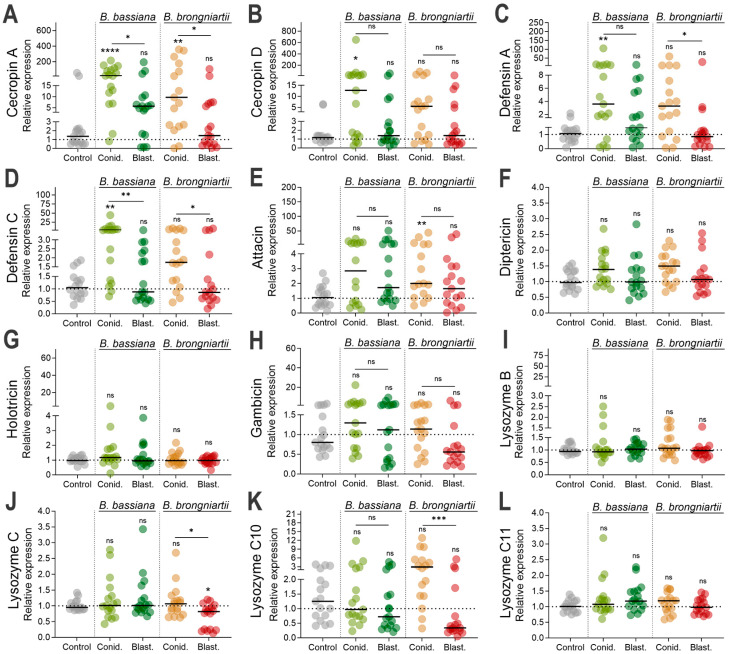
Induction of *Ae. aegypti* antimicrobial peptides at 3 d post-infection with either conidia or blastospores from *Beauveria bassiana* MBC076 and *Beauveria brongniartii* MBC397. (**A**) Cecropin A, (**B**) Cecropin D, (**C**) Defensin A, (**D**) Defensin C, (**E**) Attacin, (**F**) Diptericin, (**G**) Holotricin, (**H**) Gambicin, (**I**) Lysozyme B, (**J**) Lysozyme J, (**K**) Lysozyme C10, (**L**) Lysozyme C11. Data represent the fold change in expression from 3 independent experiments. Conid. = conidia, Blast. = blastospores. Kruskal–Wallis test with Dunn’s post-test; * *p* < 0.05, ** *p* < 0.01, *** *p* < 0.001, **** *p* < 0.0001, ns = not significant.

We next evaluated whether downstream effectors were induced by fungal infection. We investigated the expression of several antimicrobial effectors in mosquitoes infected with either fungal entomopathogen and compared this response between propagule types. Our results indicated that among the canonical antimicrobial peptide effectors, cecropin A was significantly induced during conidial infection from both fungal strains, *B. bassiana* MBC076 (Kruskal–Wallis test, *p* < 0.0001) or *B. brongniartii* MBC397 (Kruskal–Wallis test, *p* = 0.0084) (Figure 5A). However, infections with blastospores from these same fungi did not elicit cecropin A expression. In contrast, cecropin D expression was significantly induced only during infections with *B. bassiana* MBC076 conidia (Kruskal–Wallis test, *p* = 0.038) (Figure 5B). The defensins showed similar trends but with significant induction during infections with *B. bassiana* MBC076 conidia (DEFA, Kruskal–Wallis test, *p* < 0.0141; DEFC, Kruskal–Wallis test, *p* = 0.0018) (Figure 5C,D). While the expression of DEFA and DEFC in larvae infected with *B. brongniartii* MBC397 conidia were higher than expressions resulting from blastospore infections, it was not significantly different than uninfected controls (DEFA, Kruskal–Wallis test, *p* > 0.9999; DEFC, Kruskal–Wallis test, *p* > 0.9999) (Figure 5C,D). Expression of attacin was only statistically significant during infections with *B. brongniartii* MBC397 conidia (Kruskal–Wallis test, *p* = 0.0094) (Figure 5E). Gene expression evaluations with antimicrobial peptides diptericin, holotricin, gambicin, and antimicrobial effectors lysozyme B, lysozyme C, lysozyme C10, and lysozyme C11 did not show statistically significant gene induction with any of the treatments (Figure 5F–L). However, there was a significant difference in the expression of lysozyme C10 between larvae infected with conidia versus those infected with blastospores of *B. brongniartii* MBC397; nevertheless, neither were significant relative to the control group (Figure 5K).

### 3.5. Significant Reduction in Prophenoloxidase Expression During Infections with B. brongniartii MBC397 Blastospore Propagules

**Figure 6:**
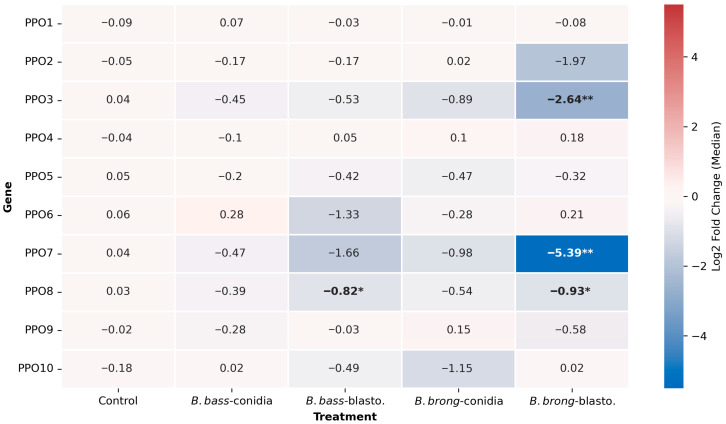
Heatmap depicting the gene expression of prophenoloxidase genes at 3d post-infection with either conidia or blastospores from *Beauveria bassiana* MBC076 or *Beauveria brongniartii* MBC397. Data represent the median fold change in expression from 3 independent experiments. Kruskal–Wallis test with Dunn’s post-test; * *p* < 0.05, ** *p* < 0.01.

To understand the implication of the phenoloxidase cascade in the larval response to fungal infection and to assess if these responses varied according to fungal propagule, we evaluated the expression of gene members of the phenoloxidase cascade (PPO1, PPO2, PPO3, PPO4, PPO5, PPO6, PPO9, and PPO10). Our assays indicated a slight decrease in the expression of PPO3, PPO5, PPO7 and PPO8 in all treatments but with a much stronger statistically significant reduction in the expression of PPO3 and PPO7 in larvae infected with *B. brongniartii* MBC397 blastospores (PPO3, Kruskal–Wallis test, *p* = 0.0233; PPO7, Kruskal–Wallis test, *p* = 0.0015) (Figure 6). In comparison, we observed a significant reduction in the expression of PPO8 in larvae treated with blastospores from either *B. bassiana* MBC076 (Kruskal–Wallis test, *p* = 0.0049) or from *B. brongniartii* MBC397 (Kruskal–Wallis test, *p* = 0.0118) (Figure 6).

## 4. Discussion

In this study, we evaluated the virulence of two entomopathogenic fungi, *Beauveria bassiana* MBC076 and *Beauveria brongniartii* MBC397, against larvae of the yellow fever mosquito *Ae. aegypti* using two types of infectious propagules: conidia and blastospores. Our results revealed that the two types of fungal propagules differed in their effectiveness against mosquito larvae and in how those larvae responded to infection.

Overall, our bioassays indicate a significant reduction in mosquito survival upon exposure to entomopathogenic fungal propagules, regardless of type. Conidial inocula, especially from *B. brongniartii* MBC 397, induced rapid and pronounced larval mortality, while blastospores resulted in more gradual mortality. This is consistent with prior studies demonstrating that the virulence of fungal propagules in aquatic environments can differ depending on species, strain, and host mosquito. For instance, Peng et al. [10] and Alkhaibari et al. [16] demonstrated faster larval mortality and higher virulence of conidia compared to blastospores of *M. anisopliae* and *M. brunneum* when used against *Ae. albopictus* and *Culex quinquefasciatus* mosquitoes, respectively. Conversely, Gomes et al. [35] and Alkhaibari et al. [8] demonstrated the higher virulence of *M. brunneum* blastospores compared to conidia against the mosquito *Ae. aegypti*. In addition, Bitencourt et al. [17] demonstrated similar virulence between conidia and blastospores of *M. anisopliae* isolates. Thus, as observed in other pathosystems, the efficacy of fungal propagules is not solely dependent on fungal species but is also influenced by propagule biology and host species. While our study did not evaluate the site of infection, previous studies using *B. bassiana* conidia as mosquito larvicidals indicated the larval gut as the primary site for fungal development [36].

Despite the differences in the timing of mortality, the overall impact of fungal infection on mosquito survival at the highest fungal concentration was similar among the treatments by day seven post-infection (our end-point of survival). However, our infection bioassays revealed interesting infection dynamics between both fungal propagules, with conidia primarily inducing mortality at the larval stage. *B. brongniartii* blastospores appear to exert their killing action at the pupal stage, with a significantly higher proportion of pupal mortality occurring approximately seven days post-infection. These findings suggest that the type of fungal propagule and fungal strain influence not only the overall mortality rate but also the progression of infection and timing of mortality. This is most likely due to their biochemical properties defining their respective infection mechanisms in an aquatic environment [26,37,38].

We also explored the larval antifungal response, observing divergent patterns of gene expression shaped by both fungal strain and propagule type. Interestingly, we observed a selective upregulation of the Toll pathway transcription factor REL1 only in larvae infected with *B. brongniartii* blastospores, while REL2 expression remained unchanged. These results suggest that fungal recognition and immune activation in mosquito larvae likely depend not only on pathogen identity but also on specific morphological and biochemical characteristics of the infecting propagule [9,10,39]. However, it is worth noting that the larval immune system undergoes substantial remodeling during metamorphosis [40,41], which can further influence immune gene activation in response to fungal challenges. For instance, bursicon homodimers were found to activate REL2 expression and to trigger the production of AMPs, thereby mediating prophylactic immunity during molting periods from larvae to early adult [42].

The expression of the antifungal effector TEP22, used here as a marker of fungal infection [30], indicates significant fungal infection by fungal conidia from *B. bassiana* and *B. brongniartii* and by blastospores from *B. brongniartii*. Our assessment of known antimicrobial effectors also revealed diverging patterns of expression depending on fungal propagule type and fungal strain. Among the ten antimicrobial effectors evaluated, only cecropin A and defensin A and C presented significant gene expression upon infection with some of the propagules. For instance, while cecropin A (CECA) was found to be significantly elicited in infections with conidia independent of fungal strain, infections with blastospores remained unchanged from controls. Along with CECA, defensin was elicited significantly during conidial infection, albeit only with infections with *B. bassiana*. Conversely, the significant elicitation of attacin during infections with *B. brongniartii* conidia, but not *B. bassiana*, could potentially indicate different mechanisms of fungal infection governing the expression of this antimicrobial peptide. Our findings align with previous studies on mosquito larvae, particularly regarding the significant upregulation of cecropin and defensin [9,16].

In contrast to the infection-modulated expression observed in adult mosquitoes [18], fungal infections in mosquito larvae failed to induce the antimicrobial effector lysozyme. This is a significant variation given that lysozyme expression was a reliable indicator of fungal infection and immune response in adult mosquitoes, with significant induction starting at the early stages of infection and in multiple mosquito species [18,43]. Our assessment also included a larva-specific lysozyme (lysozyme C10), which was found to be active at the larval and pupal stages [44], but we did not observe a significant change relative to the control group. While fungal-derived immune suppression is plausible, the absence of lysozyme induction might also reflect the developmental and tissue-specific differences in immune dynamics at the larval and adult stages. Thus, while adult mosquitoes possess a robust systemic immunity via fat body-derived effectors, the larval responses may rely more on localized epithelial defenses via antimicrobial peptides. Nevertheless, a stronger lysozyme expression has been observed in *Anopheles gambiae* larvae compared to adults [45]. Coupling this to the slight but significant downregulation of lysozyme C in mosquitoes infected with *B. brongniartii* blastospores provides further support to a fungal-derived suppression. Further studies are needed to discern the expression dynamics of this important antimicrobial effector in the mosquito larvae.

The melanization cascade is known to be an important component of the mosquito response to entomopathogenic fungal infection [22,27]. A notable observation in our study was the lack of induction of several pro-phenoloxidase genes during fungal infection and the significant downregulation of three PPO genes (PPO3, PPO7, and PPO8) in larvae exposed to *B. brongniartii* blastospores. This finding, along with the absence of antimicrobial effector induction in larvae infected with *B. brongniartii* blastospores, might indicate active immune suppression by blastospores. Similar repression of PPO3 expression has been observed in adult *Ae. aegypti* during infection with conidia from this same *B. brongniartii* strain [18], during infections with conidia from several *Cordyceps* (formerly *Isaria*) strains [29], and in the other insects infected with entomopathogenic fungi such as the moth *Spodoptera litura*, the wax worm *Galleria mellonella*, and the common fruit fly *Drosophila melanogaster* [46,47]. PPO3 was previously found to be involved in the antifungal immune response [28]. The lack of PPO gene regulation with fungal infection might also suggest that these PPO genes play diverging roles in response to fungal infection at this mosquito developmental stage. Another important observation was the selective downregulation of PPO8 expression in larvae infected with blastospores but no conidia from either fungal strain. This suggests a propagule-specific modulation of the host immune system, in particular the melanization pathway. This might reflect a combination of rapid host penetration, reduction in immune activation due to the thin-walled cell layer characteristic of blastospores [22], and blastospore-specific secretion of secondary metabolites that downregulate PPO gene expression [47,48]. Alternatively, these may also be due to distinct infection routes. For instance, a route-dependent modulation of phenoloxidase activity was reported in *Tribolium* exposed to *B. bassiana*, where fungal infection modulates phenoloxidase activity in a route-specific manner, with a strong PO response with pricking but weak activation when infection occurs via oral exposure [49].

Additionally, the potential for blastospore infection to access the hemocoel via the gut epithelium or other non-cuticular routes should be considered. *Beauveria bassiana* is known to possess genes that could potentially enable per os infection, including genes homologous to bacterial toxins (e.g., Cry-like proteins) used by pathogens infecting via the oral route [50]. This alternative entry route could help explain why blastospore infections in our study elicited minimal AMP or PPO activation, particularly for *B. brongniartii*, and may contribute to delayed mortality concentrated in the pupal stage.

The contrasting gene expression patterns observed between blastospore and conidial infections are likely related to differences in their primary routes of entry, the physiological structure of each propagule, and the early stages of host–pathogen interaction. Research by Bitencourt et al. [9] indicated that both propagule types utilize the digestive tract as a route of infection. This ingestion or direct entry route may bypass certain cuticle-associated defenses, instead engaging gut epithelial immunity and systemic hemocyte-mediated responses [9]. Regarding immune elicitation, the higher production of AMPs observed in our study during conidial infection (but not during blastospore exposure), might be linked to the absence in blastospores of the thick hydrophobic outer coat characteristic of conidia. Similar patterns have been reported at the cellular immunity level, with granulocytes and oenocytoid numbers increasing significantly upon exposure to conidia compared to blastospores [9]. These findings reinforce the importance of considering fungal propagule biology not only in terms of virulence but also in how they interact with and potentially circumvent specific immune defenses.

In summary, our study reveals that fungal virulence against mosquito larvae is shaped by complex interactions between fungal strain, propagule type, and host immune response. While conidia consistently produced higher larvicidal activity, blastospores exhibited potentially distinct immunosuppressive characteristics and delayed mortality. This underscores the importance of selecting the appropriate propagule, along with formulations, in microbial biopesticide development. Our findings also provide new insights into the mosquito immune repertoire at the larval stage in response to entomopathogenic fungal challenge. By dissecting the interplay between fungal propagule type and host immune responses, this study supports the development of fungal biopesticides by characterizing the most virulent fungal forms and host immune biomarkers to enhance the effectiveness of mosquito larval control strategies. More importantly, results suggest that the effective control of mosquitoes could be achieved by applying conidia and blastospores of certain fungi species to target both larval and pupal stages of the mosquitoes.

## Figures and Tables

**Table 1:** Calculated LC50 (7 dpi) on the highest fungal isolate dose (conidia/blastospore per mosquito) used against *Aedes aegypti* larvae.

Fungal Isolate	Propagule Type	LC_50_ (95% CI)
*B. bassiana* (MBC-076)	conidia	4.2 × 10^7^ (1.0 × 10^7^–1.8 × 10^8^)
*B. bassiana* (MBC-076)	blastospores	2.0 × 10^8^ (2.0 × 10^7^–2.1 × 10^9^)
*B. brongniartii* (MBC-397)	conidia	1.7 × 10^7^ (5.3 × 10^6^–5.4 × 10^7^)
*B. brongniartii* (MBC-397)	blastospores	8.5 × 10^8^ (6.0 × 10^7^–1.3 × 10^10^)

## Data Availability

The original contributions presented in this study are included in the article/Supplementary Material. Further inquiries can be directed to the corresponding author.

## Supplementary Materials

The following supporting information can be downloaded at https://www.mdpi.com/article/10.3390/jof11080608/s1, Table S1: Percent blastospore production; Table S2: Primer sequences used in this study (Excel) [51,52,53,54]; Table S3: Median survival. Table S4: larvae and pupae mor-tality proportions following fungal infections with conidia and blastospores; Table S5: End point survival at 7 dpi; Figure S1: Dot plot gene expression data for phenoloxidase genes.

## References

[1] Thomas M.B., Read A.F. (2007). Can fungal biopesticides control malaria?. Nat. Rev. Microbiol..

[2] Alkhaibari A.M., Carolino A.T., Bull J.C., Samuels R.I., Butt T.M. (2017). Differential Pathogenicity of *Metarhizium* Blastospores and Conidia Against Larvae of Three Mosquito Species. J. Med. Entomol..

[3] Blanford S., Shi W., Christian R., Marden J.H., Koekemoer L.L., Brooke B.D., Coetzee M., Read A.F., Thomas M.B. (2011). Lethal and pre-lethal effects of a fungal biopesticide contribute to substantial and rapid control of malaria vectors. PLoS ONE.

[4] Achonduh O.A., Tondje P.R. (2008). First report of pathogenicity of *Beauveria bassiana* RBL 1034 to the malaria vector, *Anopheles gambiae* s.l. (Diptera; Culicidae) in Cameroon. Afr. J. Biotechnol..

[5] Blanford S., Jenkins N.E., Read A.F., Thomas M.B. (2012). Evaluating the lethal and pre-lethal effects of a range of fungi against adult *Anopheles stephensi* mosquitoes. Malar. J..

[6] Chandler D. (2016). Chapter 5—Basic and Applied Research on Entomopathogenic Fungi A2. Microbial Control of Insect and Mite Pests.

[7] Butt T.M., Coates C.J., Dubovskiy I.M., Ratcliffe N.A., Lovett B., Leger R.J. (2016). Chapter Nine—Entomopathogenic Fungi: New Insights into Host–Pathogen Interactions. Advances in Genetics.

[8] Alkhaibari A.M., Carolino A.T., Yavasoglu S.I., Maffeis T., Mattoso T.C., Bull J.C., Samuels R.I., Butt T.M. (2016). *Metarhizium brunneum* Blastospore Pathogenesis in *Aedes aegypti* Larvae: Attack on Several Fronts Accelerates Mortality. PLoS Pathog..

[9] de Oliveira Barbosa Bitencourt R., Corrêa T.A., Santos-Mallet J., Santos H.A., Lowenberger C., Moreira H.V.S., Gôlo P.S., Bittencourt V.R.E.P., da Costa Angelo I. (2023). *Beauveria bassiana* interacts with gut and hemocytes to manipulate *Aedes aegypti* immunity. Parasites Vectors.

[10] Peng T.L., Syazwan S.A., Hamdan R.H., Najwa N.S., Ramli M.F., Harshiny N., Ishak I.H. (2024). Virulence and proteomic responses of *Metarhizium anisopliae* against *Aedes albopictus* larvae. Pestic. Biochem. Physiol..

[11] Charnley A.K. (2003). Fungal pathogens of insects: Cuticle degrading enzymes and toxins. Adv. Bot. Res..

[12] Jaronski S.T., Mascarin G.M. (2016). Chapter 9—Mass Production of Fungal Entomopathogens A2. Microbial Control of Insect and Mite Pests.

[13] Maldonado-Blanco M.G., Lizzette G.-S.J., Gabriela F.-P., Francisco S.-C.C., Elías-Santos M. (2014). Effect of culture medium on the production and virulence of submerged spores of *Metarhizium anisopliae* and *Beauveria bassiana* against larvae and adults of *Aedes aegypti* (Diptera: Culicidae). Biocontrol Sci. Technol..

[14] Greenfield B.P., Peace A., Evans H., Dudley E., Ansari M.A., Butt T.M. (2015). Identification of *Metarhizium* strains highly efficacious against *Aedes*, *Anopheles* and *Culex* larvae. Biocontrol Sci. Technol..

[15] Riba G., Keita A., Soares G.G., Ferron P. (1986). Comparative studies of *Metarhizium anisopliae* and *Tolypocladium cylindrosporum* as pathogens of mosquito larvae. J. Am. Mosq. Control Assoc..

[16] Alkhaibari A.M., Lord A.M., Maffeis T., Bull J.C., Olivares F.L., Samuels R.I., Butt T.M. (2018). Highly specific host-pathogen interactions influence *Metarhizium brunneum* blastospore virulence against *Culex quinquefasciatus* larvae. Virulence.

[17] Bitencourt R.D.O.B., Santos-Mallet J.R.D., Lowenberger C., Ventura A., Gôlo P.S., Bittencourt V.R.E.P., Angelo I.D.C. (2023). A Novel Model of Pathogenesis of *Metarhizium anisopliae* Propagules Through the Midguts of *Aedes aegypti* Larvae. Insects.

[18] Ramirez J.L., Dunlap C.A., Muturi E.J., Barletta A.B.F., Rooney A.P. (2018). Entomopathogenic fungal infection leads to temporospatial modulation of the mosquito immune system. PLoS Neglected Trop. Dis..

[19] Ramirez J.L., Muturi E.J., Flor-Weiler L.B., Vermillion K., Rooney A.P. (2020). Peptidoglycan Recognition Proteins (PGRPs) Modulates Mosquito Resistance to Fungal Entomopathogens in a Fungal-Strain Specific Manner. Front. Cell. Infect. Microbiol..

[20] Tawidian P., Rhodes V.L., Michel K. (2019). Mosquito-fungus interactions and antifungal immunity. Insect Biochem. Mol. Biol..

[21] Shin S.W., Kokoza V., Bian G., Cheon H.M., Kim Y.J., Raikhel A.S. (2005). REL1, a homologue of *Drosophila dorsal*, regulates toll antifungal immune pathway in the female mosquito *Aedes aegypti*. J. Biol. Chem..

[22] Lu H.L., Leger R.J. (2016). Insect Immunity to Entomopathogenic Fungi. Adv. Genet..

[23] Ramirez J.L., Muturi E.J., Barletta A.B.F., Rooney A.P. (2019). The *Aedes aegypti* IMD pathway is a critical component of the mosquito antifungal immune response. Dev. Comp. Immunol..

[24] Dong Y., Morton J.C., Ramirez J.L., Souza-Neto J.A., Dimopoulos G. (2012). The entomopathogenic fungus *Beauveria bassiana* activate toll and JAK-STAT pathway-controlled effector genes and anti-dengue activity in *Aedes aegypti*. Insect Biochem. Mol. Biol..

[25] Ramirez J.L., Hampton K.J., Rosales A.M., Muturi E.J. (2023). Multiple mosquito AMPs are needed to potentiate their antifungal effect against entomopathogenic fungi. Front. Microbiol..

[26] Qu S., Wang S. (2018). Interaction of entomopathogenic fungi with the host immune system. Dev. Comp. Immunol..

[27] Yassine H., Kamareddine L., Osta M.A. (2012). The mosquito melanization response is implicated in defense against the entomopathogenic fungus *Beauveria bassiana*. PLoS Pathog..

[28] Wang Y., Jiang H., Cheng Y., An C., Chu Y., Raikhel A.S., Zou Z. (2017). Activation of *Aedes aegypti* prophenoloxidase-3 and its role in the immune response against entomopathogenic fungi. Insect Mol. Biol..

[29] Ramirez J.L., Muturi E.J., Dunlap C., Rooney A.P. (2018). Strain-specific pathogenicity and subversion of phenoloxidase activity in the mosquito *Aedes aegypti* by members of the fungal entomopathogenic genus *Isaria*. Sci. Rep..

[30] Wang Y.H., Hu Y., Xing L.S., Jiang H., Hu S.N., Raikhel A.S., Zou Z. (2015). A Critical Role for CLSP2 in the Modulation of Antifungal Immune Response in Mosquitoes. PLoS Pathog..

[31] Joubert D.A., Walker T., Carrington L.B., De Bruyne J.T., Kien D.H.T., Hoang N.L.T., Chau N.V.V., Iturbe-Ormaetxe I., Simmons C.P., O’Neill S.L. (2016). Establishment of a *Wolbachia* Superinfection in *Aedes aegypti* Mosquitoes as a Potential Approach for Future Resistance Management. PLoS Pathog..

[32] Dzaki N., Ramli K.N., Azlan A., Ishak I.H., Azzam G. (2017). Evaluation of reference genes at different developmental stages for quantitative real-time PCR in *Aedes aegypti*. Sci. Rep..

[33] Livak K.J., Schmittgen T.D. (2001). Analysis of Relative Gene Expression Data Using Real-Time Quantitative PCR and the 2−ΔΔCT Method. Methods.

[34] Bell A.S., Blanford S., Jenkins N., Thomas M.B., Read A.F. (2009). Real-time quantitative PCR for analysis of candidate fungal biopesticides against malaria: Technique validation and first applications. J. Invertebr. Pathol..

[35] Gomes S.A., Carolino A.T., Teodoro T.B.P., Silva G.A., Bitencourt R.d.O.B., Silva C.P., Alkhaibari A.M., Butt T.M., Samuels R.I. (2023). The Potential of *Metarhizium anisopliae* Blastospores to Control *Aedes aegypti* Larvae in the Field. J. Fungi.

[36] Miranpuri G.S., Khachatourians G.G. (1991). Infection sites of the entomopathogenic fungus *Beauveria bassiana* in the larvae of the mosquito *Aedes aegypti*. Entomol. Exp. Appl..

[37] Valero-Jiménez C.A., Faino L., Spring in’t Veld D., Smit S., Zwaan B.J., van Kan J.A.L. (2016). Comparative genomics of *Beauveria bassiana:* Uncovering signatures of virulence against mosquitoes. BMC Genom..

[38] Mascarin G.M., Iwanicki N.S.A., Ramirez J.L., Delalibera Í., Dunlap C.A. (2021). Transcriptional Responses of *Beauveria bassiana* Blastospores Cultured Under Varying Glucose Concentrations. Front. Cell. Infect. Microbiol..

[39] Butt T.M., Greenfield B.P., Greig C., Maffeis T.G., Taylor J.W., Piasecka J., Dudley E., Abdulla A., Dubovskiy I.M., Garrido-Jurado I. (2013). *Metarhizium anisopliae* pathogenesis of mosquito larvae: A verdict of accidental death. PLoS ONE.

[40] Wang Z., Lu A., Li X., Shao Q., Beerntsen B.T., Liu C., Ma Y., Huang Y., Zhu H., Ling E. (2011). A systematic study on hemocyte identification and plasma prophenoloxidase from *Culex pipiens quinquefasciatus* at different developmental stages. Exp. Parasitol..

[41] Zhang J., Huang W., Yuan C., Lu Y., Yang B., Wang C.-Y., Zhang P., Dobens L., Zou Z., Wang C. (2017). Prophenoloxidase-Mediated Ex Vivo Immunity to Delay Fungal Infection after Insect Ecdysis. Front. Immunol..

[42] Zhang H., Dong S., Chen X., Stanley D., Beerntsen B., Feng Q., Song Q. (2017). Relish2 mediates bursicon homodimer-induced prophylactic immunity in the mosquito *Aedes aegypti*. Sci. Rep..

[43] Ramirez J.L., Schumacher M.K., Ower G., Palmquist D.E., Juliano S.A. (2021). Impacts of fungal entomopathogens on survival and immune responses of *Aedes albopictus* and *Culex pipiens* mosquitoes in the context of native *Wolbachia* infections. PLoS Neglected Trop. Dis..

[44] Ursic Bedoya R.J., Mitzey A.M., Obraztsova M., Lowenberger C. (2005). Molecular cloning and transcriptional activation of lysozyme-encoding cDNAs in the mosquito *Aedes aegypti*. Insect Mol. Biol..

[45] League G.P., Estévez-Lao T.Y., Yan Y., Garcia-Lopez V.A., Hillyer J.F. (2017). *Anopheles gambiae* larvae mount stronger immune responses against bacterial infection than adults: Evidence of adaptive decoupling in mosquitoes. Parasites Vectors.

[46] Bali G.K., Kaur S., Kour B.G. (2013). Phenoloxidase activity in haemolymph of *Spodoptera litura* (Fabricius) mediating immune responses challenge with entomopathogenic fungus, *Beauveria bassiana* (Balsamo) Vuillmin. J. Entomol. Zool. Stud..

[47] Matskevich A.A., Quintin J., Ferrandon D. (2010). The Drosophila PRR GNBP3 assembles effector complexes involved in antifungal defenses independently of its Toll-pathway activation function. Eur. J. Immunol..

[48] Feng P., Shang Y., Cen K., Wang C. (2015). Fungal biosynthesis of the bibenzoquinone oosporein to evade insect immunity. Proc. Natl. Acad. Sci. USA.

[49] Rafaluk-Mohr C., Wagner S., Joop G. (2018). Cryptic changes in immune response and fitness in *Tribolium castaneum* as a consequence of coevolution with *Beauveria bassiana*. J. Invertebr. Pathol..

[50] Mannino M.C., Huarte-Bonnet C., Davyt-Colo B., Pedrini N. (2019). Is the Insect Cuticle the only Entry Gate for Fungal Infection? Insights into Alternative Modes of Action of Entomopathogenic Fungi. J. Fungi.

[51] Pan X., Zhou G., Wu J., Bian G., Lu P., Raikhel A.S., Xi Z. (2012). Wolbachia induces reactive oxygen species (ROS)-dependent activation of the Toll pathway to control dengue virus in the mosquito *Aedes aegypti*. Proc. Natl. Acad. Sci. USA.

[52] Barletta A.B., Silva M.C., Sorgine M.H. (2012). Validation of *Aedes aegypti* Aag-2 cells as a model for insect immune studies. Parasites Vectors.

[53] Xiao X., Liu Y., Zhang X., Wang J., Li Z., Pang X., Wang P., Cheng G. (2014). Complement-related proteins control the flavivirus infection of *Aedes aegypti* by inducing antimicrobial peptides. PLoS Pathog..

[54] Ramirez J.L., Souza-Neto J., Torres Cosme R., Rovira J., Ortiz A., Pascale J.M., Dimopoulos G. (2012). Reciprocal tripartite interactions between the *Aedes aegypti* midgut microbiota, innate immune system and dengue virus influences vector competence. PLoS Neglected Trop. Dis..

