# Supplementary figures and images for: Larvicidal and Immunomodulatory Effects of Conidia and Blastospores of Beauveria bassiana and Beauveria brongniartii in Aedes aegypti
Source: J Fungi (Basel). 2025 Aug 21;11(8):608. doi: 10.3390/jof11080608 (PMC12387533; doi:10.3390/jof11080608)

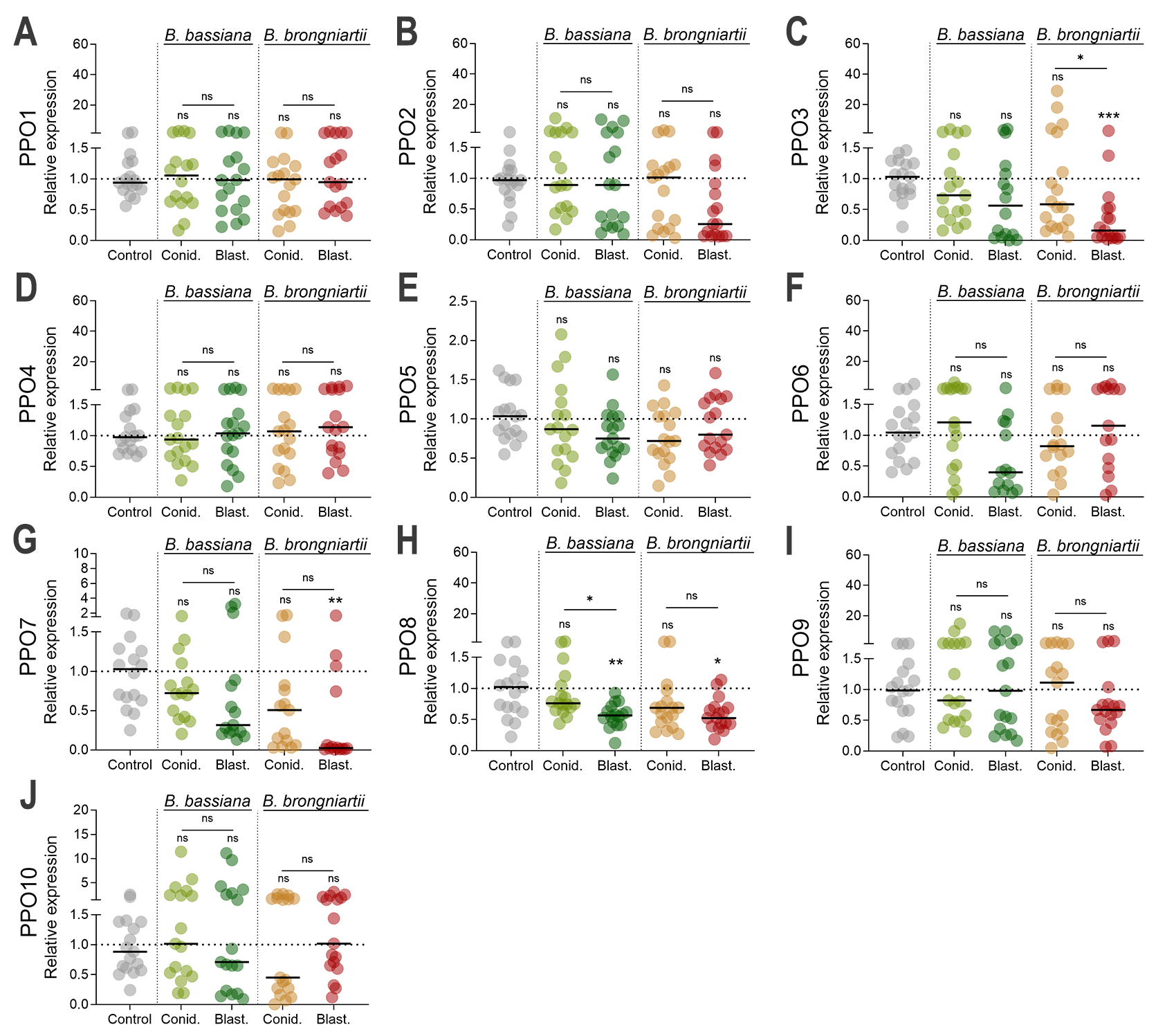

Supplement: Supplementary file 1 [file jof-11-00608-s001.zip › jof-3748759-Figure S1.tif]
